# Influenza Virus-like Particle Containing Two Different Subtypes of Hemagglutinin Confers Protection in Mice Against Lethal Challenge With A/PR8 (H1N1) and A/HK (H3N2) Viruses

**DOI:** 10.5812/ircmj.6252

**Published:** 2013-01-05

**Authors:** Farhad Rezaei, Abbas Mirshafiey, Shohreh Shahmahmoodi, Zabihollah Shoja, Nastaran Ghavami, Talat Mokhtari-Azad

**Affiliations:** 1 Department of Virology, School of Public Health, Tehran University of Medical Sciences, Tehran, IR Iran; 2 Department of Immunology, School of Public Health, Tehran University of Medical Sciences, Tehran, IR Iran

**Keywords:** Orthomyxoviridae, Virus-like particles, Influenza Vaccines

## Abstract

**Background:**

Preventing the seasonal or pandemic outbreak of influenza can be powerful and cost-effective.

**Objectives:**

In this study, we constructed a novel virus-like particle (VLP) platform that contains two hemagglutinin (HA) subtypes and evaluated immunogenicity of constructed VLP in mice.

**Materials and Methods:**

This recombinant candidate vaccine model resulted in the expression of two HAs of H1N1 and H3N2 subtypes co-localized within a VLP. Following infection of insect cells with recombinant baculovirus co-expressing H1, H3 and M1 proteins, VLPs with size of 80–120 nm were self-assembled, budding, and released into the insect culture medium. The resulting VLPs which contained two different subtypes of hemagglutinin were purified by ultracentrifugation. The immunogenicity of VLPs was evaluated in mice following immunization.

**Results:**

Our data showed that vaccination using VLPs elicited robust levels of serum IgG, and viral neutralizing antibodies against A/PR8 (H1N1) and A/HK (H3N2) viruses. Following challenge with lethal dose of A/PR8 (H1N1) and A/HK (H3N2, vaccinated mice were protected, displaying no sign of weight loss and mortality compared to non-vaccinated control mice.

**Conclusions:**

VLPs can serve as a promising vaccination strategy to control influenza virus.

## 1. Background

Influenza is a serious respiratory disease which affects human health and is lethal. The influenza virus easily spreads as an aerosol and causes acute viral respiratory disease. Seasonal influenza epidemics solitarily account for five million severe cases worldwide (1-3). In order to prevent the seasonal or pandemic outbreak of influenza, vaccination is a cost-effective tool (4, 5) and protection against influenza virus is primarily derived by antibodies induced against viral hemagglutinin (HA). In addition of being a major surface glycoprotein, HA is responsible for the attachment and penetration of viral particles into the cells during the initial stages of infection (3, 6, 7). Successful prophylactic influenza vaccines can elicit efficient antibodies against HA, which can bind to the virus and inhibit early events of the influenza infection (8). Different types of influenza vaccines are available including subunit, attenuated and inactivated influenza vaccines. The latter is the most widely used in the commercial scale (9-11). The major substrate for the preparation of inactivated influenza vaccines is embryonated chicken’s egg (12). In currently used approaches, predominant circulating strains selected by WHO/CDC are grown in chicken eggs, chemically inactivated and semi-purified (13). Regardless of being potent immunogen, the development of these vaccines takes several months due to the identification and selection of a high yield reassorted virus strain Other drawbacks include vaccines allergic to egg proteins and difficulties in supplementing embryonated eggs particularly in case of emergency or the time when the selected influenza strain is lethal to birds (1, 14). In recent years several different approaches have been applied along with conventional egg-based influenza vaccine methods. Recombinant non-infectious virus-like particles (VLPs) producing in baculovirus system, structurally naïve and immunologically relevant viral antigens, seems to be a promising and novel technology for high-yielding, safe and low-cost commercial vaccines for influenza virus (1-4, 8, 11, 15-19). Several different constructs of VLPs containing either M1-HA or M1-HA-NA have been already constructed. Influenza Matrix protein (M1) plays a pivotal role in assembly and release of Virions. Additionally, contact between the cytoplasmic tails of the influenza virus membrane proteins (HA and NA) and Matrix protein is also important for formation of the budding particle (20, 21). Based on this phenomenon, different kinds of VLPs have been approached by different research groups (1-4, 8, 12, 15-19).

## 2. Objectives

This study was aimed to construct a VLP containing both H1 and H3 on its surface as a bivalent influenza vaccine against H1N1 and H3N2 strains.

## 3. Materials and Methods

### 3.1. Cloning of H1, H3, and M1 Genes

For the construction of the VLPs, viral RNA was extracted from A/PR8 and A/HK influenza viruses (viruses were taken as a gift from *WHO, NIMR, London*) using High pure Viral RNA kit (Roche Applied Science, Mannheim, Germany) followed by Reverse transcription, polymerase chain reaction (RT-PCR) with specific oligonucleotide primers and a SuperScript® III One-Step RT-PCR System with Platinum® *Taq* High Fidelity kit (Invitrogen, USA). The produced Fragments were then visualized on 1% agarose gel at the lengths of 0.8 and 1.7 KB for M1 and HAs (H1 and H3), respectively. The primer pairs containing Restriction Sites for the amplification of the H1, H3, and M1 are listed as follows:

F/H1-PR38 5'- CGGGATCCATGAAGGCAAACCTACTGGTCC -3'

R/H1-PR38 5'-GGGGTACCTCAGATGCATATTCTGCACTGCAA-3'

H3/F –HK 5'-GCTCTAGAATGAAGACCATCATTGCTTTGAGC-3'

H3/R –HK 5'-GGGGTACCTCAAATGCAAATGTTGCACC-3'

M1/F 5'-GGAATTCATGAGTCTTCTAACCGAGGTCG-3'

M1/R 5'-GCTCTAGATCACTTGAAYCGYTGCATCTGCAC-3'

Then the products were sequenced directly using BigDye® Terminator v3.1 Cycle Sequencing Kit and a 3130 Genetic Analyzer Automated Sequencer as specified by applied biosystems protocols (Foster City, CA). The nucleotide sequences were found to be identical to the sequences published in GenBank with accession numbers: EF467821, CY044261, and CY044302.

The PCR-amplified genes of H1, H3, and M1 containing restriction sites *BamHI-KpnI, XbaI-KpnI*, and *EcoRI-XbaI*, respectively, were cloned into pFastBac1 bacmid transfer vector (Invitrogen, USA) 8. Then a bacmid transfer vector expressing both HAs and M1 genes was prepared by sub-cloning pH3 and pH1 into pM1 using restriction enzymes *SnaBI,HpaI*, and *AvrII* as Pushko et al. showed 8. This resulted in a plasmid, pM1H3H1 encoding M1, H3, and H1 genes in which the expression cassette contains a polyhedrin promoter and transcription termination signals. Following transformation of *E. coli* DH10Bac competent cells with the constructed recombinant vector genes finally transposed into the bacmid by homologous recombination as described in the pFastBac1 protocol (Invitrogen, USA).

### 3.2. Transfection of SF9 Cells by Bacmid

The recombinant bacmid DNA was transfected into 0.8-1×10^6^ of the Sf9 cells seeded in 6-well plates using CellFectin II reagent (Invitrogen, USA). The resulting recombinant baculovirus (rBV) collected from the culture medium on day 3 post-transfection as described by manufacturer (Invitrogen, USA) and stored at 4˚C.

### 3.3. Infecting SF9 Cells With rBV Expressing H1, H3, and M1 for VLP Production

To produce Influenza VLPs containing H1, H3, and M1 the Sf9 insect cells were seeded at a density of 2×10^7^ per flask and infected with recombinant baculovirus (rBV) expressing the H1, H3, and M1 proteins at MOI (multiplicity of infection) of 3. Three days post-infection, culture supernatants containing the VLPs were harvested and clarified by low-speed centrifugation (2000 rpm for 20 min at 4°C), followed by ultracentrifugation at 27,000 rpm for 4hrs to pellet. The deposited VLPs were then suspended again in PBS at 4°C overnight and further purified by a 20–30–60% discontinuous sucrose gradient at 27,000 rpm for 5hrs at 4°C.

### 3.4. SDS-PAGE and Western blot Analysis

A sodium dodecyl sulfate (SDS) 10% polyacrylamide gel and western blot analysis were used to verify the proteins content of the VLPs. 3-5ug of purified VLPs was loaded into the gel and then transferred onto a nitrocellulose membrane with the Mini Trans-Blot (Bio–Rad, CA). Following blotting, the membrane was blocked by 5 % skimmed milk solution over night at 4˚C and then incubated with H1, H3, and M1 mouse IgG mAbs (1:1000 v/v for each mAb) at room temperature. Subsequently, the membrane was incubated with goat anti-mouse IgG HRP-conjugated (1:10000 v/v, Sigma) before development.

### 3.5. Sandwich Capture ELISA

To confirm whether both hemagglutinin proteins of two subtypes of influenza virus are present on the surface of the VLPs, a sandwich capture ELISA was performed. The monoclonal Abs (mAb) used for this purpose gifted from *WHO, NIMR, London*. The H1 mAb was coated on high sorb 96 well ELISA strips (Nunc, Denmark) overnight in 4˚C followed by blocking with skimmed mike 5%. 20ug/ml VLP was added into the wells and incubated at 37˚C for 1 hour. After three consecutive washing with PBS containing 0.05% of Tween 20, H3 mAb was then added and incubated for 1 hour at 37˚C. Subsequently after four consecutive washing, anti IgG mouse HRP conjugated was added and incubated at 37°C for 1 hour. Following final wash, TMB substrate solution was added to each well and absorbance read out at 495 nm.

### 3.6. Indirect Immunofluorescence Assay

To confirm the expression and co-localization of recombinant H1and H3 proteins on the surface of sf9 cells, we performed indirect immunofluorescence assay (IIFA) using infected cells fixed with cold acetone and specific mAbs for H1, H3, and M1 proteins.

### 3.7. Haemagglutination Assays

For this purpose, suspensions of purified VLPs, influenza virus A/PR8 and A/HK with a protein concentration of 1 mg/ml were prepared in PBS. Two-fold Serial dilutions of 1ug of VLPs and the influenza viruses were prepared in 96-well plates and incubated at room temperature for 30-60 min with 0.5% guinea pig red blood cells. The HA titer was finally inspected visually and the highest dilution capable of agglutinating red blood cells was determined.

### 3.8. Haemagglutination Inhibition Assays

Serum samples were separately treated with receptor destroying enzyme (RDE) prior to being tested with a final serum dilution of 1:10. Sera were serially diluted 2-fold in microtiter plates. An equal volume of A/PR8 and A/HK viruses adjusted to approximately 4 HAU/25 ul was added to each well, separately. The plates were incubated at room temperature for 60 min before the addition of 0.5% guinea pig erythrocytes in PBS, mixed by agitation, and left for 1 h at 25˚C to allow the RBCs settle. The reciprocal of the last dilution which contained non-agglutinated RBC was considered as HAI titer. Positive and negative serum controls included in each plate. 4

### 3.9. Electron Microscopy

Coated grids of VLP sample adsorbed to the surface was stained with 1% phosphotungstic acid for 1-2 min. Excess stain wicked away with a piece of paper and then air dried for 1-3 minutes. Influenza VLPs were then visualized by using a ZEISS EM900 transmission electron microscope at 80 kV with magnification 85 kX.

### 3.10. Immunization, Challenges, and Evaluation of Humoral Immune Responses

Female BALB/c mice (10 per group) aged 6-8 weeks were used in this study. In the first experiment, 10 female BALB/c mice per group were inoculated intranasally (i.n.) with the candidate vaccine containing a total amount of 10 ug of VLP per dose two times on days 0 and 21. Two Negative control groups of BALB/c mice only received PBS instead of VLPs. Blood samples were taken before the primary and after booster inoculations by retro orbital plexus puncture at days 0 and 35, then sera were collected and serum antibodies were measured using HAI as well as ELISA. For second experiment on days 0 and 21, 10ug of influenza VLPs were intramuscularly (i.m.) inoculated in the left hind legs of 10 female BALB/c mice per group and negative control mice (mocks) received PBS only. Before virus challenge, serum samples were collected and tested by ELISA for the presence of anti HA-specific IgG antibody. ELISA plates were coated with the inactivated influenza A/PR8 and A/HK viruses, followed by the addition of goat anti-mouse IgG conjugated to horseradish peroxidase (Sigma) to detect bound antibodies. Titers were considered as the highest dilution that yielded on optical density. At the end of experiments mice were injected with 100 mg/kg intra-peritoneal ketamine and sacrificed at day 14 post-challenge.

### 3.11. Statistical Analysis

Statistical analysis of data was performed using the one way-ANOVA. P-values less than 0.05 were considered statically significant.

## 4. Results

### 4.1. Expression and Characterization of Proteins

**Figure 1:**
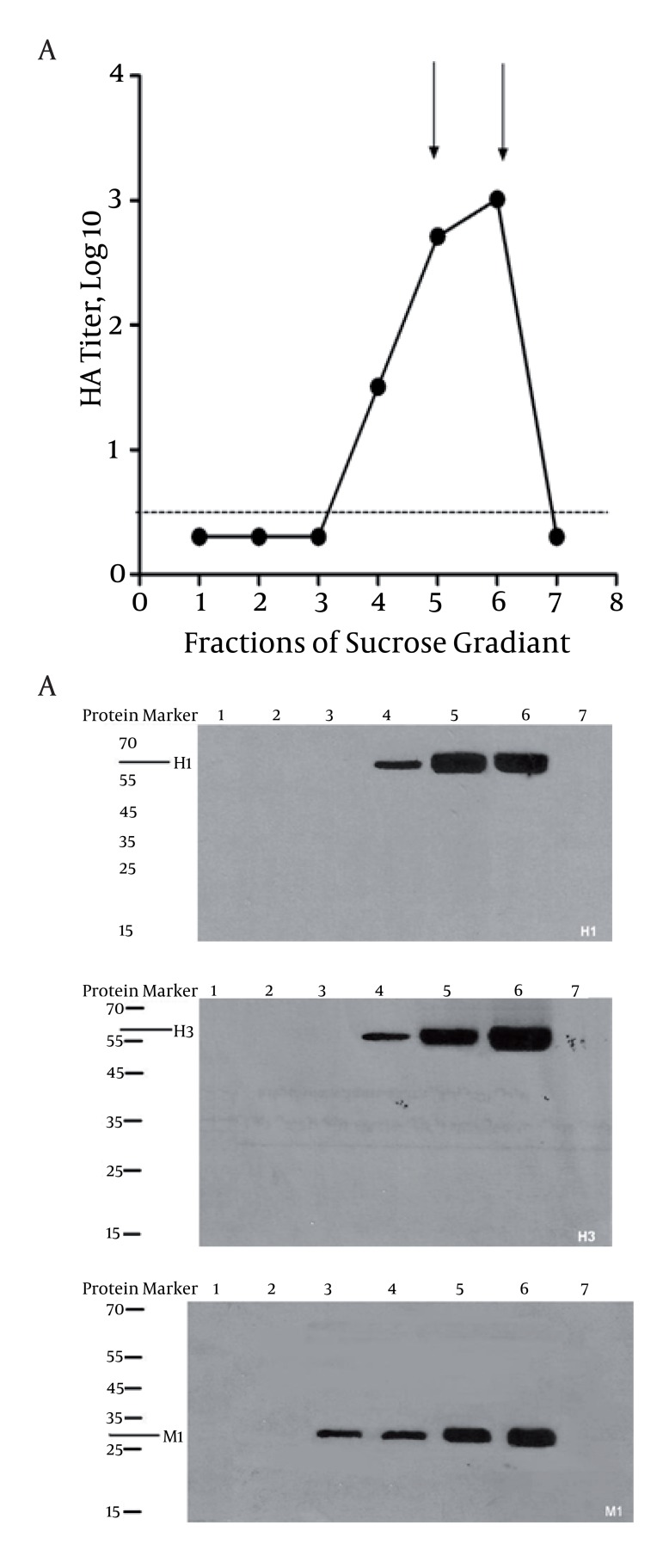
A) Fractions of Sucrose Gradient showing different logs of HA titer. Seven fractions were collected from sucrose gradient followed by haemagglutination assay. As indicated, the highest titers were found for fractions 5 and 6 as highlighted in arrows. Dash line indicates the base line. B) Immunobloting of different fractions was performed to determine the presence of H1, H3, and M1 in VLPs as illustrated

Recombinant baculovirus expressing influenza M1, H1/HA (A/PR/8/34, H1N1), and H3/HA (A/Hong Kong/68, H3N2) were generated by using the pFastbac bacmid transfer as mentioned above. To confirm the expressing of HAs (H1 and H3) and M1 protein in the insect cell by rBV, cell lysates were subjected to SDS and Western blot analysis with monoclonal antibodies specific for HAs and M1. HA proteins (H1 and H3) expressed in Sf9 cells represented a polypeptide of approximately 63 kDa indicating an uncleaved HA0 recombinant protein, as expected. The M1 protein was also represented as a major polypeptide of approximately 29 kDa (Figure 1).

### 4.2. Formation, Purification and Composition of VLPs

The Sf9 insect cells were infected with rBV expressing both HAs (H1 and H3) and M1 proteins at MOI of 3. Three days post-infection, VLPs were observed to release VLPs into the cell culture supernatant followed by purification from via a 20–30–60% discontinuous sucrose gradient as described above. In the final preparation, western blot was used to assess the presence of HAs (H1 and H3) and M1 proteins. As indicated in Figure 1B, two major fractions, located in lanes 5 and 6 found to contain both M1 and HAs. Similarly, the highest haemagglutination activity (1:2048) was observed in the fractions 5 and 6 (Figure 1A). The HA titers were 512 and 1024 per 1ug of VLPs and homologous viruses, respectively. However, we found that the HA titer of VLPs was approximately a twofold titer lower than those of influenza virions. As indicated in previously published data, the content of HA in influenza virion is estimated to be 29% of total protein (12, 19, 22, 23). Accordingly, influenza virus contains 2.9ug HA per 10ug total protein; therefore, our generated VLPs were estimated to contain 1.8ug HA per 10ug of total VLPs protein. M1 protein was also identified in fraction 3, indicating the presence of self-assembled VLPs which contain just M1 protein with no HAs glycoproteins on the surface.

### 4.3. Co-localization of Recombinant Hemagglutinins on Surface of SF9 Infected Cells

**Figure 2:**
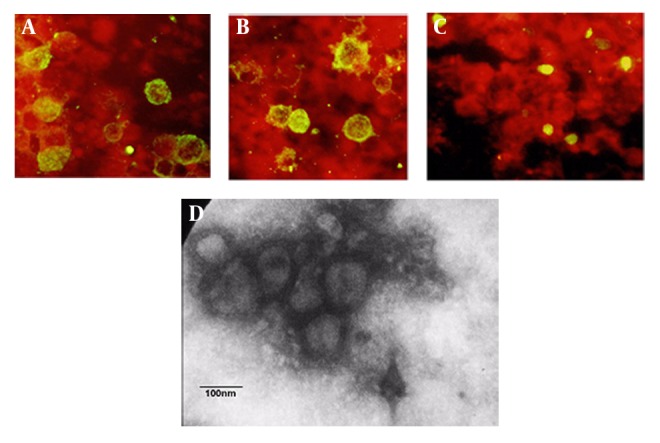
Determination of hemagglutinin and matrix proteins was performed by indirect immunofluorescence of SF9 cells infected with baculoviral expressing H1 (A), H3 (B), and M1(C). Negative staining of electron microscopy shows the influenza VLPs comprised of the H1, H3, and M1 proteins (D). Bar represents 100 nm

To further confirm the co-localization of recombinant HAs (H1 and H3) on the surface of the infected insect cells, we performed indirect immunofluorescence assay by individual mAb specific to each protein on a same preparation of the fixed SF9 cell co-expressing H1, H3, and M1 proteins. As shown in Figure 2, the recombinant glycoproteins H1 and H3 were incorporated mostly into the cell surface while M1 proteins remained almost cytoplasmic. (Green: sf9 cells expressing proteins, Red: sf9 cells not expressing protein). We also used non-infected sf9 cell as a negative control (data not shown). In addition, randomly taken electron microscopy micrographs showed pleomorphic VLPs of different sizes of 80-120 nm range (Figure 2D).

### 4.4. Verification of H1 and H3 Glycoproteins on the Surface of VLPs

**Figure 3:**
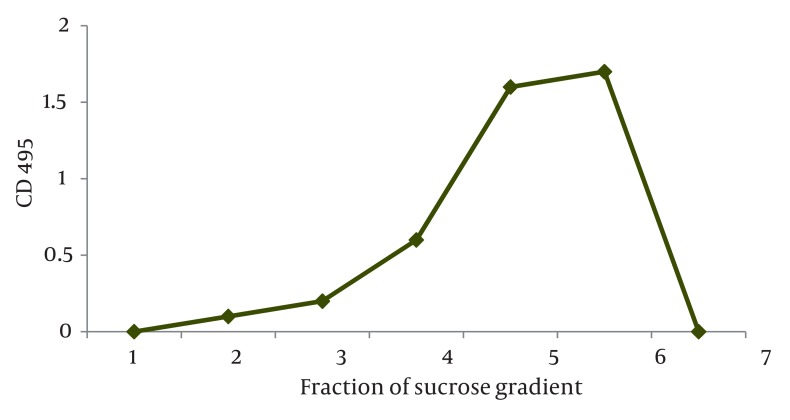
Concomitant presence of H1 and H3 glycoproteins on the surface of influenza VLPs. The graph shows the presence of both H1 and H3 glycoprotein types after performing a qualitative sandwich capture ELISA using specific H1 and H3 mAb. Similar to the titers obtained by HA assay, the highest OD was found for fractions 5 and 6 as indicated in arrows

To confirm the presence of both HA protein (H1 and H3) types on the surface of VLPs, we performed a triplicate sandwich capture ELISA on the recovered fractions from the sucrose gradient as described in section 3.11. Existent H1 glycoproteins on the surface of VLP were captured by H1 mAb coated on the wells of ELISA plate and VLPs not included the H1 protein, were excluded after washing step. In the next step, H3 mAb was added to each well and only the captured VLPs which contained the H3 glycoprotein in their context bound to the H3 mAb. Following the addition of HRP-conjugated goat anti-mouse IgG, and chromogen substrate the absorbance was determined at 450 nm. In addition to negative control, H1N1 and H3N2 viruses were used as antigen controls. We found the highest OD in the fraction 5 and 6 of recovered VLPs from sucrose gradients. All together, the results showed the concomitant presence of the glycoproteins on the surface of VLPs as illustrated in Figure 3.

Based on these data, infecting insect cells with rBV expressing HA (H1 and H3) and M1 proteins resulted in the expression and self-assembly of VLPs that were secreted from infected cells into Based on these data, infecting insect cells with rBV expressing HA (H1 and H3) and M1 proteins resulted in the expression and self-assembly of VLPs that were secreted from infected cells into the culture medium. The resulting VLPs; therefore, retained haemagglutination functions of both H1 and H3 subtypes.

### 4.5. Antibody Responses, HAI and Neutralizing Antibody

**Figure 4:**
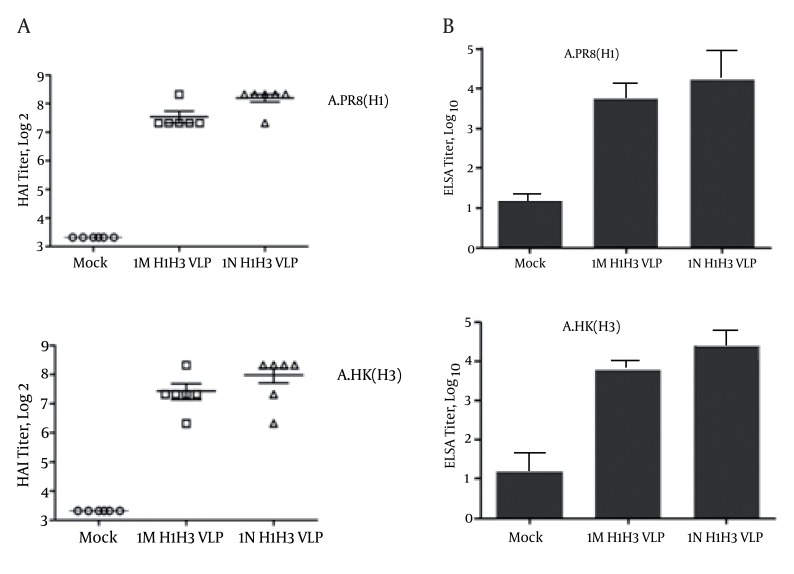
Protective immune responses induced by bivalent influenza VLP vaccines. Different groups of mice were immunized intranasally and intramuscularly with 10ug of influenza VLPs containing H1 and H3 glycoproteins. At week 5, all mice inoculated with the two doses of H1H3 VLP (10ug per dose). HAI titers and the levels of IgG antibodies specific to the corresponding homologous strains A/PR8 (H1N1) and A/HK (H3N2) are shown in Figure 4A and 4B, respectively. (IN and IM are abbreviation for intranasally and intramuscularly vaccination routes). (Results are significant at p<0.05, Error bars refer to mean)

The current study aims to evaluate the protective immune responses which were induced by bivalent influenza VLP candidate vaccine. Looking at the pre-existing antibodies to currently circulating human influenza viruses, all mice were negative (HAI ≤ 10) prior to immunization. 10ug of influenza H1H3VLP was used to immunize groups of mice intranasally and intramuscularly. Following two immunizations, the H1H3VLP candidate vaccine induced HAI antibodies against the homologous strains. At week 5, all mice inoculated with the two doses (10 ug H1H3VLP per dose) had an HAI response with titers ranging from 1:80 to 1:320 against virus strains. We observed the high rise of HAI in the group of mice immunized intranasally (1:320) whereas the titer of HAI in mice immunized intramuscularly was 1:160 (P ≤ 0.05). In mice that received a mock immunization we could not detect (< 1:10 titer) HAI activity against any of the H1N1 and H3N2 antigens as shown in Figure 4A. Additionally, the levels of IgG antibodies specific to the corresponding homologous strains A/PR8 (H1N1) and A/HK (H3N2) were determined by ELISA on plates coated with inactivated influenza viruses (Figure 4B). The groups of mice which received bivalent influenza VLPs also showed high levels of IgG antibody specific to both viruses. Mice immunized intranasally with H1H3VLPs induced higher levels of specific IgG antibodies than those immunized intramuscularly (p ≤ 0.05). Both H1 and H3 glycoproteins present in the influenza VLPs showed to be immunogenic and in terms of inducing specific immune responses against the homologous virus strains, the H1H3-VLP candidate vaccine resembles current influenza vaccines.

### 4.6. Bivalent Influenza VLPs Survived Lethal Influenza Virus Challenges

**Figure 5:**
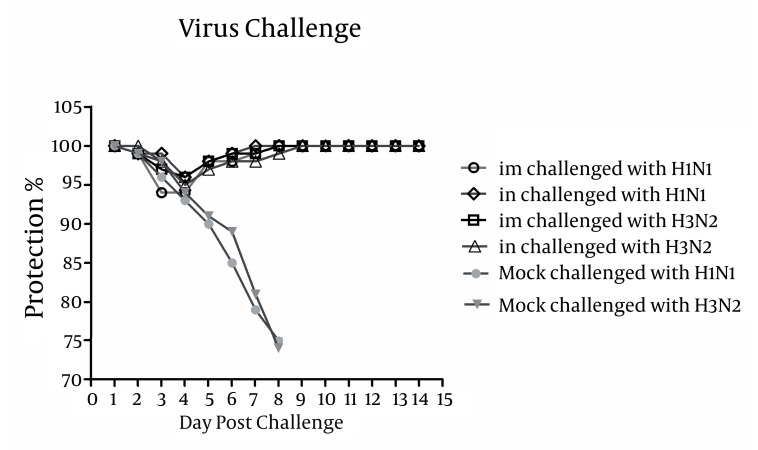
Protection of influenza H1H3 VLP-vaccinated mice following lethal virus challenges with of A/PR8 (H1N1) and A/HK (H3N2). Mice were monitored daily for two weeks to check body weight loss and percentage of survival. All VLP-immunized mice were survived lethal virus challenges with A/PR8 (H1N1) and A/HK (H3N2) and none of them showed signs of illness or weight loss. In contrast, mock-immunized controls started showing signs of illness, weight loss and morbidity at days 3 post-challenge

To determine whether immunized mice are protected against a lethal challenge, influenza H1H3-VLP immunized mice were challenged with lethal doses of A/PR8 (H1N1) and A/HK (H3N2) as illustrated in Figure 5. All H1H3 VLP-immunized mice were survived lethal virus challenges with A/PR8 (H1N1) and A/HK (H3N2) and none of them showed signs of illness or weight loss. By contrast, 3 days post-challenge mock-immunized mice controls were found to show signs of infection and illness. Concurrently these mice showed significant weight loss (P ≤ 0.05) and by day 6 they lost approximately 25% of their original body weight and all died by 8 days post-challenge, suggesting the applicability of H1H3-VLPs in the context of protection.

## 5. Discussion

Annually, emergence of new subtypes of influenza virus among human population is a great challenging issue. Lack of enough immunity of a given society to emerged subtypes is of great public health concern and yet vaccination remains a powerful and most economically public health strategy against both seasonal and pandemic influenza (10, 24-26). Considering a candidate as part of influenza vaccine strategy, the development of influenza VLPs in insect cells has been suggested to overcome several drawbacks attributed to the egg-based system such as possible disruption of vaccine supplies due to the shortage of fertilized chicken embryos or potential low-yield in the case of producing highly pathogenic influenza viruses. Furthermore, the lack of viral genomic RNAs or DNAs makes VLPs as a non-infectious and safe tool for broad applications. In addition, the recombinant baculovirus expression system offers high yields of influenza VLPs and it has shown that the amount of purified VLPs via this system is approximately as the same amount of purified influenza virus obtained from egg allantoic fluid (27).

In the current study, we presented novel VLPs containing HAs from two different subtypes of influenza virus (H1 and H3). Using H1N1 and H3N2 influenza viruses, we have already shown requirements for the assembly of VLPs and their potential in influenza VLP vaccine candidate. As consistent with previously published data (1-4, 8, 12, 15-19), they demonstrates the generation of VLPs via recombinant baculovirus system expressing M1 and HAs proteins of influenza A/PR8 (H1N1) and A/HK (H3N2) viruses. The assembly of VLP are likely depend on M1 protein as its significance has been previously highlighted in both virus assembly and budding process (8, 20, 21). Both the size and the morphology of influenza VLPs bored resembling to their corresponding virions, suggest accurate particle geometry and architecture (8). The variation seen in the size of VLPs was found to be similar to the previous observations of pleomorphic influenza virions (1-3, 8, 12).

In order to improve the coverage and the protection, the chimeric VLP candidate vaccine was developed by using two subtypes of HAs from different influenza strains. As formalin inactivated viruses may carry non-native HA proteins, the effectiveness of immune responses against these proteins can be reduced (3). Comparing to inactivated viruses, the HA glycoproteins expressed on the surface of VLPs are unmodified and native in conformation and therefore not affected by fixative. Due to the native three-dimensional conformation of expressed antigens, a chimeric influenza VLP may be more immunogenic than inactivated influenza vaccine. Our data also suggest a beneficial role of antigen sparing effects by using two proteins on the surface on influenza VLPs. However further experiments are needed to evaluate and compare the efficacy of VLP and other individual proteins as vaccines. In this study, bivalent influenza H1H3-VLPs were administered to mice intramuscularly and intranasally. Similar to the conventional influenza vaccine, the protective efficacy of the VLPs candidate vaccines depends mostly on the induction of neutralizing antibodies against the HA protein as shown here and by others (1-6, 8, 28). Accordingly, the neutralizing antibody responses were induced against the individual components of bivalent influenza VLPs. Our results indicate the bivalent influenza VLPs as an alternative influenza candidate vaccine. Influenza VLPs can also induce protective immunity comparable to the conventional influenza vaccine (9).

All together, the current study provides further evidence supporting the production of influenza VLP via insect cells as an alternative and promising approach to the egg-based conventional influenza vaccine. Additionally, this study showed that immunization with bivalent-VLPs can trigger the same type of protection induced by the current standard inactivated vaccines. The VLP approaches have the advantage by which their manufacture can overcome the necessity for embryonated chicken eggs. In the current study, we investigated the application of just two HA subtypes so far; however, in order to improve of neutralizing immunity against different subtypes, it may be possible to increase the number of HA strains included in a multivalent VLP format (1-4, 28). Considering the fact that influenza VLPs can be assembled from only structural proteins, it may be also possible to develop a chimeric VLP containing three or more HA glycoproteins of influenza or glycoproteins from other viruses such as parainfluenza and respiratory syncytial virus through utilizing baculoviruses or other alternative methods like DNA and virus vectors (8).
